# Investigation of retroviral integration preference via high-throughput sequencing

**DOI:** 10.1186/1753-6561-6-S6-P22

**Published:** 2012-10-01

**Authors:** Matthew C LaFave, Gaurav K Varshney, Shawn M Burgess

**Affiliations:** 1 Genome Technology Branch, National Human Genome Research Institute, National Institutes of Health, Bethesda, MD 20892, USA

## 

The integration of retroviral DNA into the host genome is a key step of the retrovirus lifecycle. The positions of such integrations are non-random and are characteristic to the virus in question. Retroviral vectors have been useful in gene therapy, but the approach carries with it the danger of host gene misregulation by viral integration, potentially contributing to cancer. An understanding of how integration sites are determined is critical for any approach seeking to avoid such consequences. To better understand the factors influencing retroviral integration site selection, we are generating integrations of murine leukemia virus (MLV) in human K562 cells. We use massively parallel Illumina technology to sequence the cellular genome and have developed a bioinformatic pipeline to efficiently identify sites of integration. Our aim is to create a high-resolution, high-density integration map with 1 million unique integrations mapped to single-nucleotide resolution. This map will identify regions prone or refractory to MLV integration. We plan to compare this map with data from the ENCODE Consortium, looking for significant associations with characteristics of the local genomic environment, to enhance our understanding of MLV integration site selection.

